# Design of Wearable Breathing Sound Monitoring System for Real-Time Wheeze Detection

**DOI:** 10.3390/s17010171

**Published:** 2017-01-17

**Authors:** Shih-Hong Li, Bor-Shing Lin, Chen-Han Tsai, Cheng-Ta Yang, Bor-Shyh Lin

**Affiliations:** 1 Department of Thoracic Medicine, Chang Gung Memorial Hospital at Linkou, Taoyuan 33305, Taiwan; penbus504@gmail.com; 2 Department of Computer Science and Information Engineering, National Taipei University, New Taipei City 23741, Taiwan; bslin@mail.ntpu.edu.tw; 3 Institute of Imaging and Biomedical Photonics, National Chiao Tung University, Tainan 71150, Taiwan; melon0511@hotmail.com; 4 Department of Thoracic Medicine, Chang Gung Memorial Hospital at Taoyuan, Taoyuan 33378, Taiwan; yang1946@cgmh.org.tw; 5 Department of Respiratory Therapy, College of Medicine, Chang Gung University, Taoyuan 33302, Taiwan

**Keywords:** airway obstruction, wheeze detection, short-term breathing sound, spectral integration

## Abstract

In the clinic, the wheezing sound is usually considered as an indicator symptom to reflect the degree of airway obstruction. The auscultation approach is the most common way to diagnose wheezing sounds, but it subjectively depends on the experience of the physician. Several previous studies attempted to extract the features of breathing sounds to detect wheezing sounds automatically. However, there is still a lack of suitable monitoring systems for real-time wheeze detection in daily life. In this study, a wearable and wireless breathing sound monitoring system for real-time wheeze detection was proposed. Moreover, a breathing sounds analysis algorithm was designed to continuously extract and analyze the features of breathing sounds to provide the objectively quantitative information of breathing sounds to professional physicians. Here, normalized spectral integration (NSI) was also designed and applied in wheeze detection. The proposed algorithm required only short-term data of breathing sounds and lower computational complexity to perform real-time wheeze detection, and is suitable to be implemented in a commercial portable device, which contains relatively low computing power and memory. From the experimental results, the proposed system could provide good performance on wheeze detection exactly and might be a useful assisting tool for analysis of breathing sounds in clinical diagnosis.

## 1. Introduction

Abnormal breathing sounds (such as crackles, rhonchus, and wheezing sounds) are usually considered as indicator symptoms in chronic respiratory diseases [1], such as chronic obstructive pulmonary disease (COPD), chronic bronchitis, and bronchial asthma, etc. For these diseases, unnecessary secretions (such as sputum) would be produced in the respiratory tract and then cause chronic inflammation leading to airway obstruction. When the state of airway obstruction acutely exacerbates, the inside diameter of airway will be narrowed, and the smooth muscle in the outer wall of the airway will also be tightened during breathing [2]. Therefore, the airflow velocity will be changed when the air flows from the normal airway into the narrowing airway, producing abnormal breathing sounds, such as wheezes [3]. In the clinic, a wheezing sound is a kind of continuously abnormal breathing sound with a specific tone [4], and it is usually considered as an indicator symptom to reflect the degree of airway obstruction [5]. When the wheezing sound occurs, the patient might have no sufficient amount of air to maintain normal breathing, resulting in dyspnea, asphyxia or other life-threatening situations [6]. Therefore, it is important to detect wheezing sounds automatically, and then to provide prompt medical treatment for patients with acute airway obstruction.

Currently, investigation of breathing sounds is mainly based on the auscultation approach by the experienced physicians. This method is simple, convenient, and non-invasive, but it is also subjectively dependent on the experience of the physicians, the variability of the human auditory system and the specifications of stethoscopes [7,8]. In previous studies [9,10,11,12,13,14,15,16,17,18,19,20], several approaches, such as a spirometer, vibration response image (VRI), piezoelectric sensor and air-coupled microphone, had also been used to evaluate the feature of breathing sounds objectively. In 1994, Schreur et al. determined the parameters of lung sound intensity (LSI), frequency content in quartile point and peak frequency from the flow-dependent power spectrum of lung sounds to investigate the lung sound features of patients with bronchial asthma [15]. In 1999, Kiyokawa et al. investigated the feature pattern of hourly nocturnal wheezing count (NWC), the duration of wheezing sounds, and the forced expiratory volume in one second (FEV_1_), which was obtained from a spirometer to evaluate the severity of bronchoconstriction [16]. In 2002, Gross et al. used the ratio of relative power at maximal flow (RPMF) between inspiratory and expiratory in the frequency band of 300–600 Hz to investigate the lung sound features of pneumonia [17]. In 2014, Içer et al. used the ratio of maximum and minimal frequency in power spectral density (PSD), and the normalized instantaneous frequency of lung sounds to distinguish different abnormal lung sounds [18]. Most of the above methods just provide the lung sound information in frequency or time domain, respectively. In 2008, Sello et al. compared three quartile frequencies in the global wavelet spectrum to investigate the severity of respiratory insufficiency in patients with COPD [19]. In 2009, Riella et al. used image processing techniques to extract the spectral projection of lung sound spectrogram to identify wheezing sounds [20]. From the spectrogram of lung sounds, the information of lung sounds in both time and frequency domains could be extracted. However, it also requires a relatively long raw data and computational complexity for time frequency analysis, and this is inconvenient for several commercial portable devices, which contains relatively low computing ability and memory, to perform real-time wheeze detection. Moreover, most of the devices used in the above studies were not wearable, and were also not convenient for long-term monitoring of breathing sounds in daily life.

In order to improve the above issues, a wearable breathing sound monitoring system for real-time wheeze detection was proposed in this study. In this proposed system, a wearable breathing sound acquisition module was designed to collect breathing sounds wirelessly in daily life. A breathing sound analysis algorithm was also proposed in this study. By using this algorithm, the spectral features of short-term breathing sounds would be real-time and continuously extracted to construct a characteristic pattern, and from the feature pattern, the information of breathing sounds in both of time and frequency domains could be effectively obtained and applied in wheeze detection. This algorithm required only lower computational complexity to perform real-time wheeze detection, and it is suitable to be implemented in a commercial portable device, which contains relatively low computing capability. Finally, the difference between features of wheezing and healthy breathing sounds was also investigated in this study.

## 2. Methods and Materials

### 2.1. System Hardware Design and Implementation

**Figure 1:**
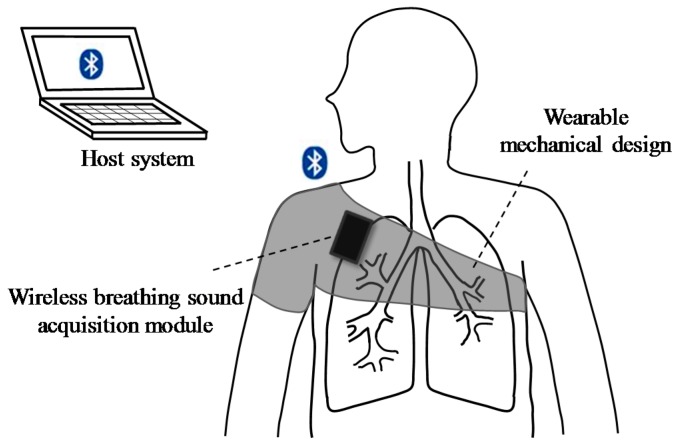
Basic scheme of proposed wearable and wireless breathing sound monitoring system.

Figure 1 shows the basic scheme of the proposed wearable and wireless breathing sound monitoring system, including a wireless breathing sound acquisition module, a wearable mechanical design, and a host system. The wireless breathing sound acquisition module is embedded into the wearable mechanical design, and it is placed on the upper right anterior chest surface of the user to acquire the breathing sound. The wearable mechanical design aims to not only embed with the proposed module, but also provide a suitable pressure on the wireless breathing sound acquisition module to maintain a good contacting condition between the acoustic sensor and the chest wall. In this mechanical design, the acoustic sensor could acquire a good signal quality of breathing sound, and users could be easy to wear and use in daily life. After the breathing sound is acquired, amplified, and digitized by the proposed module, it will then be transmitted to the host system wirelessly. Finally, the real-time breathing sound monitoring program built in the host system will receive, display, record and analyze the acquired breathing sound to provide the information of the breathing sound to the medical staff.

#### 2.1.1. Wireless Breathing Sound Acquisition Module

**Figure 2:**
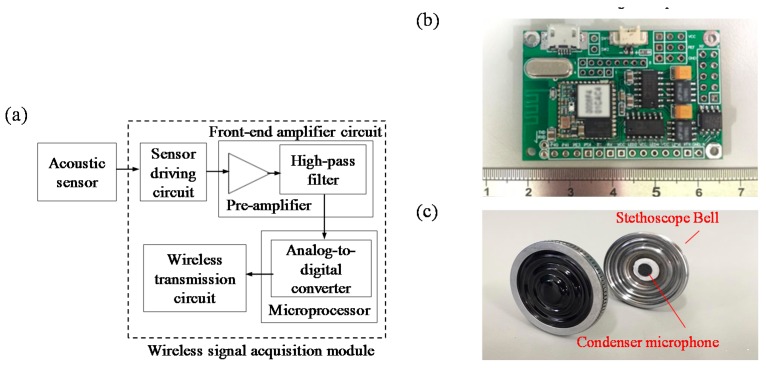
(**a**) Block diagram and (**b**) photograph of proposed wireless breathing sound acquisition module and (**c**) photograph of acoustic sensor.

Figure 2a,b show the block diagram and photograph of the proposed wireless breathing sound acquisition module, and it mainly contains several parts, including an acoustic sensor, a sensor driving circuit, a front-end amplifier circuit, a microprocessor, and a wireless transmission circuit. In the wireless breathing sound acquisition module, the acoustic sensor consists of an omnidirectional condenser microphone (TS-6022A, TRANSOUND, Dongguan, China) and a stethoscope bell (Harvey™ DLX, Welch Allyn, Skaneateles Falls, New York, NY, USA), as shown in Figure 2c. It is designed to acquire breathing sound effectively, and transduced sound into an electrical signal. Here, the sensor driving circuit is intended to provide a stable driving voltage for the omnidirectional condenser microphone, and eliminate variation from the power source. Next, the signal of the breathing sound will be amplified and filtered by a front-end amplifier circuit. The total gain of the front-end amplifier circuit is set to 500 times, and the frequency band is set to > 150 Hz. Next, the pre-processed breathing sound will be digitized by a 12-bit analog-to-digital converter built in the microprocessor (MSP430, Texas Instruments, Dallas, TX, USA) with the sampling rate of 2048 Hz, and then be sent to a wireless transmission circuit, which consists of a printed circuit board (PCB) antenna and a Bluetooth module (Ct-BT02, Connectec, Taiwan) with the Bluetooth v2.0+ enhanced data rate (EDR) specification. Here, the power consumption and data rate of the wireless acquisition module are about 75 mW and 33k bps. Finally, the wireless transmission circuit will transmit the collected breathing sound to the host system wirelessly. This module is operated at 33 mA with a commercial 250 m-Ah Li-ion battery, and can continuously operate over seven hours.

#### 2.1.2. Wearable Mechanical Design

**Figure 3:**
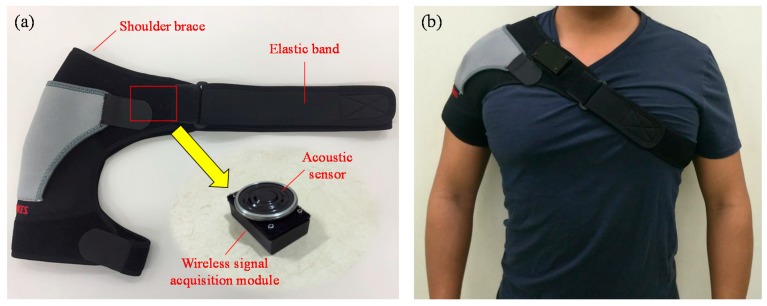
(**a**) Photograph of wearable mechanical design, and (**b**) photograph of wearing the wireless breathing sound monitoring system.

Figure 3a,b shows the photograph of the wearable mechanical design and photograph of wearing the wireless breathing sound monitoring system during experiments, and it mainly consists of a shoulder brace, an elastic band and an area of Velcro. The structure of the shoulder brace is used to embed the proposed wireless breathing sound acquisition module and also support the weight of this module. Moreover, by adjusting the tightness of elastic band, the proposed module can easily fit the chest contour of the user to maintain a good contacting condition between the acoustic sensor and the chest surface to reduce the artificial influence of motion. In this wearable mechanical design, the area of Velcro is used to fix the proposed module on the shoulder brace so that the proposed system can be easy to wear and use in daily life.

#### 2.1.3. Host System

In this study, the platform of the host system is a commercial tablet with the operation system of Window 10. Moreover, a real-time breathing sound monitoring program, developed using Microsoft C#, is also built in the host system, and this program also implements the proposed breathing sound analysis algorithm to provide the basic function of monitoring, recording, and analyzing the breathing sound.

### 2.2. Breathing Sound Analysis Algorithm

**Figure 4:**
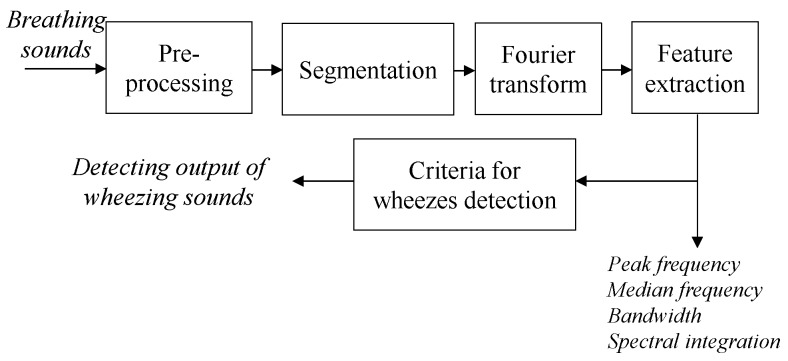
Flowchart of breathing sound analysis algorithm.

Wheezing sound is a continuously abnormal breathing sound, which is commonly disclosed in patients with COPD or other airways’ obstructive respiratory diseases. Wheezing presented with a specific characteristic in duration and frequency domain than other breathing sounds. The range of frequency of normal breathing sound evenly distributes between 100 Hz and 1000 Hz. For a wheezing sound, the frequency range is mainly between 250 Hz and 800 Hz, and it can be presented as a specific narrow line pattern, which is maintained over about 250 milliseconds [21], in the spectrogram of breathing sounds. In this study, the breathing sound analysis algorithm is designed to extract the features of breathing sound in time and frequency domains, and its flowchart is shown in Figure 4. 

Before extracting the features of breathing sounds, the received breathing sound has to be first pre-processed by a band-pass filter (frequency band: 150 Hz–1000 Hz) to reserve meaningful components of breathing sounds and also remove heart sound, muscle interference sound and blood sound [2]. Next, the raw breathing sound will be split into 250-ms breathing sound segments with 200-ms overlapping, and then the power spectrums of these breathing sound segments will be calculated by using Fast Fourier Transform with a Hanning window. After obtaining the power spectrum of each breathing sound segment, then the features of breathing sound in frequency domain can be calculated. Here, the ratios of the spectral integration (SI) features *SI*_0Hz–250Hz_ (from 0 Hz to 250 Hz), SI_250Hz–500Hz_ (from 250 Hz to 500 Hz), and SI_500Hz–1000Hz_ (from 500 Hz to 1000 Hz) to SI_0Hz–1000Hz_ (from 0 Hz to 1000 Hz) are defined as the normalized spectral integration (NSI) features NSI_0Hz–250Hz_, NSI_250Hz–500Hz_, and NSI_500Hz–1000Hz_, respectively. In this study, Fisher linear discriminant analysis (LDA) was used to separate wheezing and normal breathing sounds. After experimenting, the normalized spectral integration NSI_0Hz–250Hz_, NSI_250Hz–500Hz_, and NSI_500Hz–1000Hz_ were used as the frequency-domain features to detect the wheezing sounds, and they have better recognizing performance than other features. If the feature of breathing sound fits the following criteria:

(i) Score1 = –230.54489 + 402.72499 × ${\mathrm{NSI}}_{0\mathrm{HZ}-250\mathrm{HZ}}$ + 500.32269 × ${\mathrm{NSI}}_{250\mathrm{HZ}-500\mathrm{HZ}}$ + 677.28994 × ${\mathrm{NSI}}_{500\mathrm{HZ}-1000\mathrm{HZ}}$.
  Score2 = –266.87228 + 418.88239 × ${\mathrm{NSI}}_{0\mathrm{HZ}-250\mathrm{HZ}}$ + 554.36286 × ${\mathrm{NSI}}_{250\mathrm{HZ}-500\mathrm{HZ}}$ + 699.35894 × ${\mathrm{NSI}}_{500\mathrm{HZ}-1000\mathrm{HZ}}$.
  If Score1 < Score 2, then this segment will be recognized as an abnormal breathing sound.
(ii) If the duration of abnormal breathing sound > 250 ms [22].

**Figure 5:**
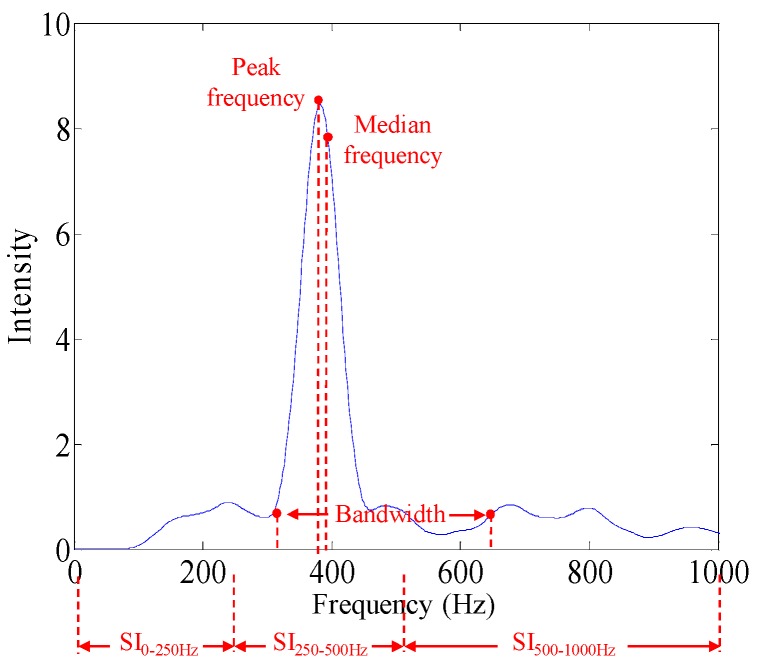
Illustration for quantitative information of breathing sounds.

Then, it will be recognized as a wheezing sound. All the quantitative information of this wheezing event, as illustrated in Figure 5, such as the peak frequency, median frequency, bandwidth, and duration, will then also be extracted and reserved. Here, the peak frequency is defined as the frequency corresponding to the highest peak in the power spectrum of the breathing sound. The median frequency is defined as the frequency that is at the center of the power spectrum of the breathing sounds. The bandwidth is defined as the frequency range between the frequencies corresponding to 25% and 75% of total integration area in the power spectrum of the breathing sound. Here, the peak frequency and median frequency are most frequently used to describe the feature of breathing sounds. Therefore, they are also extracted in this study. The changes of the peak frequency and the bandwidth will contribute to the change of the spectral integration of breathing sound directly. Moreover, the peak frequency usually shifts within a breathing cycle. Therefore, in this study, the spectral integrations of breathing sound are used as the major factors to detect wheezing sounds.

### 2.3. Experimental Design

In this study, the experimental data was collected from 40 adult patients and 11 healthy adults (males and females are 42 and 9 participants, respectively), at Chang Gung Memorial Hospital, Taiwan. The mean age of these participants was 61.9 ± 24.7 years old. The Institutional Review Board has approved this experiment (Institutional review board, No.103-3295A3), Chang Gung Memorial Hospital, Taiwan, and all participants provided an informed consent before the experiment. 

Before patients enrolling to study, physicians had to complete history taking and physical examination including breathing sounds. When a doctor disclosed wheezing via conventional stethoscope, this patient was enrolled into the study. The doctor also recorded breathing sounds at the same time. In this study, every doctor is a qualified Pulmonologist in Taiwan. They have abundant clinical practice experience. After recording breathing sounds, pulmonologists had to discuss and confirm the recorded file and voice quality first. Then, engineers analyzed voice according to study protocol and program. Finally, engineers and pulmonologists discussed final result after analysis together and made sure the data was correct. The acoustic sensor of the proposed system was placed on the right upper anterior chest wall (at the first intercostal space on the midclavicular line) to collect two-minute data of breathing sounds. In this study, every physician was a qualified pulmonologist in Taiwan. They had abundant clinical practice experiences. After recording breathing sounds, pulmonologists had to discuss and confirm the recorded file and voice quality first. Then, engineers analyzed voice according to study protocol and program. Finally, engineers and pulmonologists discussed final results after analysis together and made sure that the data was correct. Here, the analysis of variance (ANOVA) method was used to analyze the feature difference between wheezing and normal breathing sounds, and the software MATLAB (MATLAB, Math Works, Natick, MA, USA) was used to perform ANOVA. The difference significance was defined as *p* < 0.05 in this study. 

## 3. Results

### 3.1. Feature Patterns of Wheezing and Healthy Breathing Sounds

**Figure 6:**
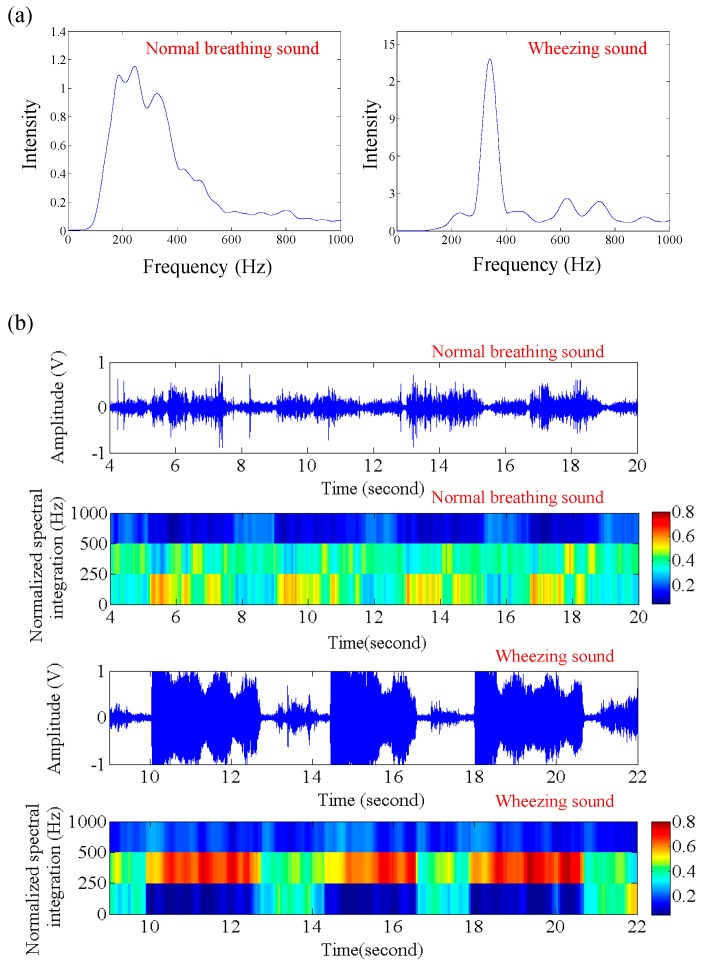
(**a**) Spectrum of wheezing and healthy breathing sounds, and (**b**) raw data and feature patterns for wheezing and healthy breathing sounds.

In this section, the spectral features of wheezing and healthy breathing sounds were investigated. Figure 6a shows the power spectrum of wheezing and healthy breathing sounds. The spectral distribution of wheezing sounds was mainly from 250 Hz to 500 Hz, and is relatively narrower than that of normal breathing sounds. Moreover, the peak intensity in the power spectrum of the wheezing sounds was also obviously larger than that of the normal breathing sounds. Figure 6b shows the time frequency feature pattern for wheezing and healthy breathing sounds. Here, the values of NSI_0Hz–250Hz_, NSI_250Hz–500Hz_, and NSI_500Hz–1000Hz_ were used as the frequency-domain feature in this feature pattern. In the feature pattern of healthy breathing sounds, the values of NSI_0Hz–250Hz_ were similar to that of NSI_250Hz–500Hz_, and greater than that of NSI_500Hz–1000Hz_. However, for the feature pattern of wheezing sounds, the values of NSI_250Hz–500Hz_ were obviously larger than that of NSI_0Hz–250Hz_ and NSI_500Hz–1000Hz_. From the difference of different feature patterns, wheezing sounds could easily be distinguished from the normal breathing sounds. Moreover, the duration of wheezing sound was about 736.86 ± 311.40 ms.

### 3.2. Feature Difference between Wheezing and Healthy Breathing Sounds

**Figure 7:**
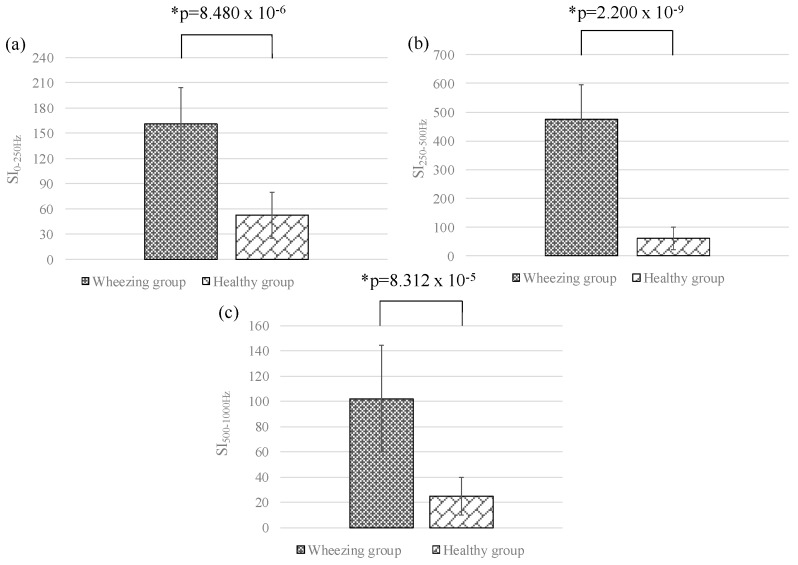
Means and standard deviations of (**a**) *SI*_0Hz-250Hz_; (**b**) *SI*_250Hz-500Hz_; and (**c**) *SI*_500Hz-1000Hz_ for different groups. Here, * denotes significant difference.

**Figure 8:**
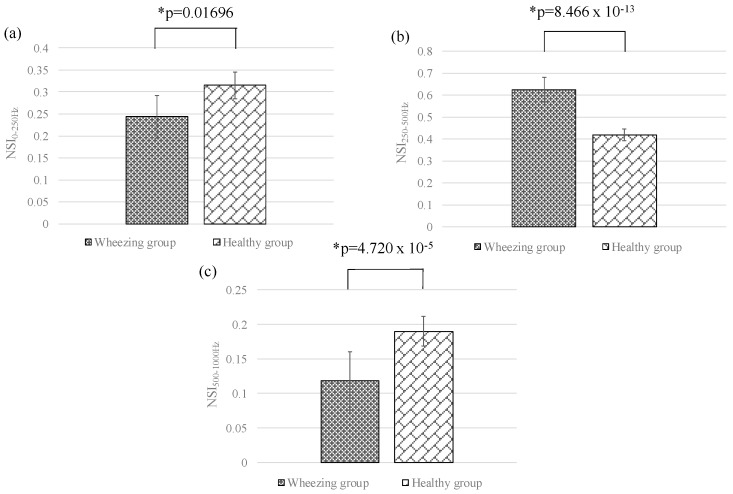
Means and standard deviations of (**a**) *NSI*_0Hz-250Hz_; (**b**) *NSI*_250Hz-500Hz_; and (**c**) *NSI*_500Hz-1000Hz_ for different groups. Here, * denotes significant difference.

**Figure 9:**
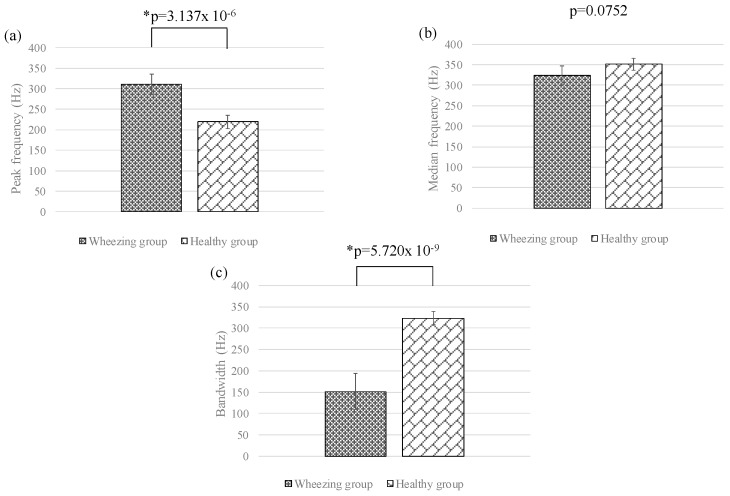
Means and standard deviations of (**a**) peak frequencies; (**b**) median frequencies; and (**c**) bandwidths for different groups. Here, * denotes significant difference.

In this section, the differences between the features of wheezing and healthy breathing sounds were investigated. Figure 7a–c shows the means and standard deviations of SI_0Hz–250Hz_, SI_250Hz–500Hz_, and SI_500Hz–1000Hz_ for different groups, respectively. The value of SI_250Hz–500Hz_ for wheezing sounds was significantly stronger than that of normal breathing sounds. However, the values of SI_0Hz–250Hz_ and SI_500Hz–1000Hz_ for wheezing sounds were similar to that of normal breathing sounds. Figure 8a–c show the means and standard deviations of NSI_0Hz–250Hz_, NSI_250Hz–500Hz_, and NSI_500Hz–1000Hz_ for different groups. For wheezing sounds, the value of NSI_250Hz–500Hz_ was obviously larger than the values of NSI_0Hz–250Hz_ and NSI_500Hz–1000Hz_. However, for normal breathing sounds, the value of NSI_0Hz–250Hz_ was similar to the value of NSI_250Hz–500Hz_, and was larger than the value of NSI_500Hz–1000Hz_. Figure 9a–c show the comparison of the peak frequencies, the median frequencies, and the bandwidths for different groups, respectively. The peak frequency of wheezing sounds was significantly higher than that of healthy breathing sounds. Moreover, the bandwidth of wheezing sounds was also significantly narrower than that of healthy breathing sounds. However, the median frequency of wheezing sounds was similar to that of healthy breathing sounds. The features for different groups were summarized in Table 1. In this study, the method of Fisher LDA was used to discriminate the two groups. The performance of using parameters of SI and NSI and the combination of SI and NSI to discriminate the two groups were compared. We found that using the parameters of NSI to discriminate the two groups could provide a better performance. Moreover, the criterion of wheezing detection was defined as the mentioned criterion in Section 2.2.

### 3.3. Performance of Breathing Sound Analysis Algorithm

In this section, the performance of detecting wheezing events by using the proposed breathing sound analysis algorithm was investigated. Before evaluating the performance of the proposed algorithm, several parameters of the binary classification test have to be defined first: true positive (TP) denotes that a real wheezing sound event was correctly detected as a wheezing sound event. False positive (FP) denotes that a non-wheezing sound event was incorrectly detected as a wheezing sound event. True negative (TN) denotes that a non-wheezing sound event was correctly detected as a non-wheezing sound event. False negative (FN) denotes that a real wheezing sound event was incorrectly detected as a non-wheezing sound event. The experimental results for the performance of detecting wheezing sound events are shown in Table 2. Here, a total number of 952 breathing sound events were used for this test. The sensitivity and positive predictive values (PPVs) of detecting wheezing sound events were about 91.51% and 100%, respectively. The false detections mainly resulted from the decayed breathing sound, the spectral shift of wheezing sound (>250 Hz), or the interference of environmental noise, such as the speech sound of the staff or families.

## 4. Discussion

For the normal breathing sounds, the value of NSI_0Hz–250Hz_ was similar to NSI_250Hz–500Hz_, and larger than NSI_500Hz–1000Hz_. Therefore, the spectral feature of healthy breathing sounds was exhibited as the characteristic of the low-pass filter due to the chest surface [23,24,25]. From the experimental results, it was shown that the value of NSI_250Hz-500Hz_ for wheezing sounds was larger than that of NSI_0Hz–250Hz_ and NSI_500Hz–1000Hz_, and this also indicated that the spectral distribution of wheezing sounds mainly concentrated at a frequency range from 250 Hz to 500 Hz due to the phenomenon of bronchoconstriction. In the previous study [26], it indicated that, for wheezing sounds, the main frequency range in the spectrum was between 200 Hz and 600 Hz, and this also fits our experimental results.

In the clinic, a wheezing sound is a kind of continuous musical sounds containing a specific tone and the sinusoidal wave appearance, resulting from the airway wall oscillation and vortex shedding in central airways [27,28,29,30,31,32]. Because of the obstructive airways, the given airflow, which goes through the narrowing bronchus, will result in the change of airflow’s velocity, and this is also associated with louder breathing sounds, and the larger peak intensity in the power spectrum [33,34,35,36]. From the experimental results in this study, the peak frequency and its intensity for wheezing sounds were higher and larger than that of normal breathing sounds (about 100 Hz–300 Hz [37]). Moreover, the bandwidth of wheezing sounds was narrower than that of normal breathing sounds. This result also fits the above-mentioned phenomenon. However, the difference of the median frequency between different groups was not obvious. The duration of wheezing sounds was about 736.86 ± 311.40 ms, and this also fits the results in previous studies (over 150 ms) [23,25]. Here, the occurrences of the FP events are mainly caused by the influence of background noise (speech voice) or artificial motion (the friction between chest wall and acoustic sensor), and that of FN events are mainly caused from the weaker amplitude or the shift of the wheezing features in frequency domain.

In previous studies, many methods have been proposed to investigate the features of various abnormal breathing sounds (such as wheezing sounds) for patients with obstructive pulmonary diseases, and the comparison between the proposed system and other methods is summarized in Table 3. Schreur et al. investigated the power spectrum to analyze the frequency features of breathing sounds in patients with asthma [15]. They indicated that even though the lung function was within the normal range, the generation or transmission of breathing sounds for the asthma group was still different from that of the healthy control group. Moreover, the quartile frequencies in the power spectrum of asthma group were higher than that of healthy control group, and they considered that wheezing sounds contained the peak frequency which was above 150 Hz and was at least three times higher than the baseline level. Uwaoma et al. developed a time frequency threshold-dependent (TFTD) algorithm to detect wheezing sounds in a smartphone [38]. The peak frequency, the number of consecutive harmonics, and the duration was used as the wheezing features, but they also indicated that the peak frequency of breathing sounds, that is highly similar to that of wheezing sounds, would easily result in the detecting failure. Jin et al. proposed a time frequency decomposition method to obtain a noise-resistant time frequency contour and detect wheezing sounds under background noise [39]. Riella et al. proposed an image processing method to extract the time frequency features of wheezing sounds from the spectral projection pattern of a spectrogram [20]. They indicated that the wheezing sounds obviously appeared as an isolated higher amplitude feature in the spectrogram. However, it might be inconvenient for real-time wheeze detection in commercial mobile phones or tablets, which may contain relatively low computing capability, due to the higher computational complexity and the requirement of longer raw data. Içer et al. extracted the frequency features from the power spectral density (PSD) based on the Welch method, and used the technique of support vector machine (SVM) to classify different abnormal breathing sounds [18]. They indicated that the ratios of the minimum and maximum frequency in PSD for rhonchus and crackles could be distinguished. Lin et al. employed the order truncate average (OTA) method to enhance the features of wheezing sounds in a spectrogram, and used the technique of back-propagation neural network (BPNN) to detect wheezing sounds [40]. Several parameters extracted from the shape feature of breathing sounds in spectrogram were used as the features of wheezing sounds. Lin et al. also used Mel frequency cepstral coefficient (MFCC) and Gaussian mixture model (GMM) to detect wheezing sounds [41]. The above method could provide a good performance of detecting wheezing sounds, but they also required more computational complexity. In Table 3, studies [20,39,40] processed spectrograms to obtain the features of wheezes, and their computational complexities are all in $O\left(n^{2}\right)$. Refs. [18,38,41] and our proposed system processed spectra from fast Fourier transform (FFT) to obtain the features of wheezes, and their computational complexities are all in O(nlogn). Thus, Refs. [18,38,41] and our proposed system have lower computational complexities than Refs. [20,39,40]. In Refs. [18,38,41], they have to use classifiers to recognize wheezes, but our proposed system only uses addition and compares with thresholds, so our proposed system has lower computational complexity than Refs. [18,38,41].

Most of the above methods only provide the simple information in frequency domain, such as peak frequency and median frequency, and this could not provide sufficient information to distinguish wheezing sounds. By using time frequency analysis and the technique of supervised learning classifier (SVM, BPNN, GMM, etc.), they could provide good performance on detecting wheezing sounds, but they also required more computational complexity. To improve the above issue, the NSI obtained from short-term breathing sounds was used to analyze the time frequency feature of breathing sounds in this study. Different from other methods, which required relatively longer raw data and more computational complexity, the proposed method could easily extract the time frequency feature from short-term breathing sounds, and required lower computational complexity. The proposed method could easily be implemented in commercial mobile phones or tablets, which may contain relatively low computing capability. Moreover, by using the wearable mechanical design and wireless communication, the proposed system could be more convenient and more free to collect the breathing sounds in daily life than other studies in Table 3.

## 5. Conclusions

We proposed a wearable and wireless breathing sound monitoring system. Here, a wireless breathing sound acquisition module and a wearable mechanical design were also designed to collect breathing sounds wirelessly in daily life. Moreover, a breathing sounds analysis algorithm, which uses the NSI obtained from short-term breathing sounds, was also proposed to analyze the time frequency feature of breathing sounds. The proposed algorithm required only short-term breathing sound data and lower computational complexity. Therefore, it is suitably implemented in commercial mobile phones or tablets, which may contain relatively low computing capability to perform real-time wheeze detection. It could also provide the objectively quantitative information of breathing sounds (peak frequency, median frequency, bandwidth, duration, and NSI) to the professional physicians. From the experimental results, the features of SI_250Hz-500Hz_, NSI_0Hz-250Hz_, NSI_250Hz-500Hz_, peak frequency, and bandwidth of wheezing sounds were significantly different from that of normal breathing sounds. Therefore, the proposed system contains the potential for being developed as a useful monitoring system for assisting with diagnosis of chronic respiratory diseases in the future.

## Figures and Tables

**Table 1:** Features in time and frequency domains for wheezing and healthy breathing sound groups.

	Wheezing Sound Group	Normal Breathing Sound Group	*p*-Value
Peak frequency (Hz)	310.52 ± 40.94	219.13 ± 49.79	* 3.137 × 10^−6^
Median frequency (Hz)	323.52 ± 36.70	350.88 ± 59.34	0.0752
Bandwidth (Hz)	151.47 ± 48.43	323.44 ± 68.85	* 5.720 × 10^−9^
*SI*_0–250Hz_	161.12 ± 64.64	52.42 ± 55.045	* 8.480 × 10^−6^
*SI*_250–500Hz_	475.38 ± 215.21	60.66 ± 50.43	* 2.200 × 10^−9^
*SI*_500–1000Hz_	101.97 ± 75.31	24.92 ± 18.65	* 8.312 × 10^−5^
*NSI*_0–250Hz_	0.243 ± 0.091	0.315 ± 0.088	* 0.01696
*NSI*_250–500Hz_	0.623 ± 0.071	0.417 ± 0.028	* 8.466 × 10^−13^
*NSI*_500–1000Hz_	0.118 ± 0.042	0.190 ± 0.046	* 4.720 × 10^−5^
Duration of wheezing sounds (milliseconds)	736.86 ± 311.40	－	－

* means significance difference (*p* < 0.05).

**Table 2:** Performance of proposed method on wheeze detection.

		Wheezing Sounds Events Detected by Proposed Algorithm		
		+	–	Total
Real breathing sound event	+	496 (TP)	46 (FN)	542
	–	0 (FP)	410 (TN)	410
	Total	496	456	952

Here, + denotes wheezing event, and – denotes non-wheezing event.

**Table 3:** System comparison between proposed system and other systems.

	R. J. Riella et al. [20]	F. Jin et al. [39]	C. Uwaoma et al. [38]	S. Içer et al. [18]	B. S. Lin et al. [40]	B. S. Lin et al. [41]	Proposed System
Breathing sounds	Wheezing sounds	Wheezing sounds	Wheezing and crackle sounds	Rhonchus and crackles sounds	Wheezing sounds	Wheezing sounds	Wheezing sounds
Sensing technique	–	Electrical condenser microphone	Smart-phone	Electronic stethoscope	Electrical condenser microphone	Electrical condenser microphone	Electrical condenser microphone
Measurement Location	–	Anterior chest	–	Six zones on posterior chest	Trachea	Trachea	Anterior chest
Feature extraction technique	Spectral projection, artificial neural network	Time-frequency decomposition, k-nearest neighbor	Time-frequency threshold dependent algorithm	PSD based on welch method, support vector machine	Order truncate average, back-propagation neural network	Mel frequency cepstral coefficient, Gaussian mixture model	Normalized spectral integration
Computational complexity	High	High	Low	Medium	High	Medium	Low
Wearable device	–	No	No	No	No	No	Yes
Wireless transmission	No	No	No	No	No	No	Yes
Applications	Wheeze detection	Wheeze detection	Wheeze detection	Analysis of abnormal breathing sound	Wheeze detection	Wheeze detection	Wheeze detection, breathing sound analysis
